# The Safety and Efficacy of a Mixture of Honey, Olive Oil, and Beeswax for the Management of Hemorrhoids and Anal Fissure: A Pilot Study

**DOI:** 10.1100/tsw.2006.333

**Published:** 2006-02-02

**Authors:** Noori S. Al-Waili, Khelod S. Saloom, Thia N. Al-Waili, Ali N. Al-Waili

**Affiliations:** Al-Waili's Foundation for Science and Trading, New York, USA

**Keywords:** honey, olive oil, beeswax, hemorrhoid, fissure, United States

## Abstract

We have found that a mixture of honey, olive oil, and beeswax was effective for treatment of diaper dermatitis, psoriasis, eczema, and skin fungal infection. The mixture has antibacterial properties. A prospective pilot study was conducted to evaluate the therapeutic effect of topical application of the mixture on patients with anal fissure or hemorrhoids.Fifteen consecutive patients, 13 males and 2 females, median age 45 years (range: 28—70), who presented with anal fissure (5 patients) or first- to third-degree hemorrhoids (4 with first degree, 4 with second degree, and 2 with third degree), were treated with a 12-h application of a natural mixture containing honey, olive oil, and beeswax in ratio of 1:1:1(v/v/v). Bleeding, itching, edema, and erythema were measured using a scoring method: 0 = none, 1 = mild, 2 = moderate, 3 = severe, and 4 = very severe. The pain score was checked using a visual analog scale (minimum = 0, maximum = 10). Efficacy of treatment was assessed by comparing the symptoms' score before and after treatment; at weekly intervals for a maximum of 4 weeks. The patients were observed for evidence of any adverse effect such as appearance of new signs and symptoms, or worsening of the existing symptoms. The honey mixture significantly reduced bleeding and relieved itching in patients with hemorrhoids. Patients with anal fissure showed significant reduction in pain, bleeding, and itching after the treatment. No side effect was reported with use of the mixture. We conclude that a mixture of honey, olive oil, and beeswax is safe and clinically effective in the treatment of hemorrhoids and anal fissure, which paves the way for further randomized double blind studies.

## INTRODUCTION

Hemorrhoidal disease and anal fissure are common disorders of the anorectal area. In addition to surgery, many procedures and preparations have been used for treatment of hemorrhoids[1,2] (Table 1). Third- and fourth-degree hemorrhoids may require hemorrhoidectomy, rubber band ligation, sclerotherapy, infrared

**TABLE 1**
**Some Interventions Used for Management of Hemorrhoids**

| | |
|---|---|
| Nonpharmacological interventions | Such as avoidance of constipation and excessive strains, high fiber intake, plenty of water and fluids, sitting in warm bath |
| Pharmacological interventions | Such as stool softeners, bulk laxative, rectal preparations, topical anesthetics, systemic analgesics, and anti-inflammatory agents |
| Protectants | Such as aluminum hydroxide gel, calamine, cod liver oil, glycerin, shark liver oil, white petrolatum, kaolin, mineral oil |
| Local anesthetics | Such as lidocaine, benzocaine, tetracaine |
| Astringents | Such as calamine, zinc oxide |
| Vasoconstrictors | Such as ephedrine sulfate, epinephrine |
| Keratolytics | Such as alcloxa, resorcinol |
| For third- and fourth-degree hemorrhoids | Rubber band ligation |
| | Infrared coagulation |
| | Injection sclerotherapy |
| | Bipolar diathermy |
| | Cryosurgery |
| | Direct current probe |
| | Hemorrhoidectomy |

photocoagulation, cryotherapy, bipolar diathermy, or laser therapy. Surgical complications include hemorrhage, urinary retention, stricture, infection, incontinence, and wound dehiscence[6]. Further, topical nifedipine and isosorbide dinitrate 1% ointment gave good results in the treatment of hemorrhoids[7,8]. A mixture of herbs, a vegetable-based hemorrhoid ointment, phenol oil, Ginko biloba-Troxerutin-Heptaminol, bioflavonoids, and dietary supplementation with micronized *Aesculus hippocastanum*, *Ruscus aculeatus*, *Centella asiatica*, and *Hamamelis virginiana* are used as alternative treatment for patients with hemorrhoids[9,10,11,12,13,14,15]. In the treatment of anal fissure, stool softeners, warm sitz baths, local anesthetic agents, corticosteroid-containing ointments, and proteolytic enzyme preparations have been used in various combinations[1]. Complete pain relief occurred with use of nitroglycerine ointment[7]. Authors recommended the use of botulinum toxin as the first therapeutic approach for patients with chronic anal fissure and risk factors for incontinence[16].

Olive oil, beeswax, and honey are natural materials that contain flavonoids, antioxidants, antibacterial and fungal ingredients; they affect cytokines production by skin cells when applied topically[17,18,19,20, 21,22]. We have demonstrated that a mixture containing honey, olive oil, and beeswax (in ratio 1:1:1 v/v/v) was effective to treat diaper dermatitis, eczema, psoriasis, and skin fungal infection[23,24]. In addition, we found that the mixture had antimicrobial properties[25]. Anal fissures and hemorrhoids are characterized by pain, itching, edema, signs of inflammation, and bleeding, and since there are no medications that cure hemorrhoids, the goal of medication is to relieve burning, discomfort, inflammation, irritation, itching, pain, soreness, and swelling[26]. The aim of the study was to investigate the effect of the mixture on the signs and symptoms of anal fissure and hemorrhoids.

## METHODS

Fifteen consecutive patients presenting with either chronic anal fissure (5 patients) or hemorrhoids (10 patients) were randomly selected for the study. There were 13 males and 2 females, median age of 45 years (range: 28–70). The target population for this pilot study was patients with first- to third-degree internal hemorrhoids or chronic anal fissure. Four patients had first-degree hemorrhoids, four patients had second-degree hemorrhoids, and two patients had third-degree hemorrhoids. Anascopy was performed to locate the degree of the grade of hemorrhoids. Patients with chronic fissure showed evidence of posterior circumscribed ulcer, sentinel tag of skin, and induration at the edges. Patients with complicated fissure, cicatricial deformation, and subfissural infiltration were excluded. Informed consent was obtained from the patients.

## Intervention and Doses

Patients were provided with the honey mixture prepared by thoroughly mixing natural honey, olive oil, and beeswax (v/v,1:1:1). The mixture was stored in a dark container and at room temperature. Mixing the same volumes of honey, olive oil, and beeswax resulted in a honey mixture that contained 50% honey, 29% olive oil, and 21% beeswax (wt/v). The honey used was dark yellow in color, of multifloral origin, and its composition included (per 100 g of honey) fructose 38 g%, glucose 28 g%, moisture 20%, acidity 13%, vitamin C 2.3 mg%, copper 0.098 mg%, zinc 0.6 mg%, sucrose 0.5 g%, and glutathione reductase 0.52 mg%. Natural olive oil prepared by cold press was used. The natural ingredients were supplied with courtesy by Dubai Specialized Medical Center, Islamic Establishment for Education, Dubai, United Arab Emirates. With use of cloves and syringes, the patients were asked to apply about a spoon size of the mixture around the skin twice daily. The patients were asked to sleep in a supine position for 15 min after application and to apply soft gauze to the anal region to keep the mixture at the site of application.

## Assessment and Statistical Analysis

The patients were asked to stop taking other medications during the trial. They were followed up at 1-week intervals for a maximum of 4 weeks for pain, bleeding, itching, edema, erythema, and side effects. The pain score was checked using a visual analog scale (minimum = 0, maximum = 10). The bleeding, itching, edema, or erythema were assessed by a scoring method: 0 = no symptoms, 1 = mild, 2 = moderate, 3 = severe, and 4 = very severe. The score was expressed as mean ± standard deviation. Patients who showed complications such as thrombosed hemorrhoids or worsened symptoms were considered as treatment failure and were dropped from follow-up. Any new sign or symptom that appeared during therapy was recorded as an adverse effect.

ANOVA was used to compare between means of groups. Probability value less than *0.05* was significant. *F test* was used to evaluate the statistical significance of between-groups differences. *F value* means measurement of distance between individual distributions. As $F$ goes up, $P$ goes down.

## RESULTS

Regarding patients with hemorrhoids, significant pain relief was achieved within 1 week and all patients were free of pain within 3 weeks of treatment (see Table 2). Significant reduction of bleeding was obtained in all the patients; except for two with hemorrhoids (pretreatment bleeding score was 2 and 3) who showed mild bleeding, all the patients had no rectal bleeding after 4 weeks (see Table 3). Itching was completely relieved within the first 2 weeks of the treatment. Erythema and edema seen in three patients with hemorrhoids completely disappeared within 2 weeks. The patients did not show relapse of piles during or after defecation after 3 weeks of treatment. The two patients with third-degree hemorrhoids scheduled for surgical intervention showed marked improvement within 4 weeks without the need for surgery.

**TABLE 2**
**Score (Mean ± SD) of Common Symptoms of Hemorrhoids and Anal Fissure and Their Response to Mixture**

| Diseases | Symptoms (Number of Patients) | Before Treatment | After Treatment 1 | 2 | 3 | 4 | F Value |
|---|---|---|---|---|---|---|---|
| Hemorrhoids | Pain (8) | 2.77 ± 2.07 | 1.62 ± 0.74 | 0.62 ± 0.51 | 0 | 0 | 16.97 |
| | Bleeding (10) | 1.6 ± 0.69 | 0.7 ± 0.67 | 0.7 ± 0.67 | 0.5 ± 0.52 | 0.2 ± 0.42 | 7.35 |
| | Itching (7) | 1.57 ± 0.78 | 0.42 ± 0.78 | 0 | 0 | 0 | 13.10 |
| Anal fissure | Pain (5) | 6 ± 1.58 | 2.2 ± 1.1 | 0.6 ± 0.54 | 0 | 0 | 40.17 |
| | Bleeding (5) | 2.2 ± 0.83 | 1.2 ± 0.83 | 0.8 ± 0.44 | 0 | 0 | 13.25 |
| | Itching (4) | 1.5 ± 0.57 | 0.75 ± 0.5 | 0 | 0 | 0 | 14.14 |

**TABLE 3**
**Scores of the Signs and Symptoms Encountered in the Patients with Hemorrhoids Before and 4 Weeks After Treatment with the Mixture**

| Number of Patients | Pretreatment Score | | | | | Score after 4 Weeks of Treatment | | | | |
|---|---|---|---|---|---|---|---|---|---|---|
| | Pain | Bleeding | Itching | Edema | Erythema | Pain | Bleeding | Itching | Edema | Erythema |
| 1 | 6 | 3 | 2 | 2 | 1 | 0 | 1 | 0 | 0 | 0 |
| 2 | 7 | 2 | 3 | 1 | 1 | 0 | 1 | 0 | 0 | 0 |
| 3 | 2 | 2 | 1 | 0 | 0 | 0 | 0 | 0 | 0 | 0 |
| 4 | 4 | 2 | 0 | 0 | 0 | 0 | 0 | 0 | 0 | 0 |
| 5 | 3 | 1 | 0 | 0 | 0 | 0 | 0 | 0 | 0 | 0 |
| 6 | 3 | 1 | 0 | 0 | 0 | 0 | 0 | 0 | 0 | 0 |
| 7 | 2 | 1 | 1 | 0 | 0 | 0 | 0 | 0 | 0 | 0 |
| 8 | 1 | 2 | 1 | 0 | 0 | 0 | 0 | 0 | 0 | 0 |
| 9 | 0 | 1 | 2 | 1 | 1 | 0 | 0 | 0 | 0 | 0 |
| 10 | 0 | 1 | 1 | 0 | 0 | 0 | 0 | 0 | 0 | 0 |

Patients with anal fissure showed significant reduction in pain, bleeding, and itching after the treatment. Signs of healing such as regression of posterior ulcer and epithelization were evident in all the patients within the 4 weeks of treatment. Erythema and edema seen in two patients with anal fissure completely disappeared within 2 weeks. Side effects were not noticed with use of the mixture in any patient recruited and no complications were experienced either.

## DISCUSSION

The results of the study demonstrated that the mixture of natural honey, olive oil, and beeswax was effective for management of anal fissure and hemorrhoids. The improvement was probably due to the antimicrobial, anti-inflammatory, antioxidant, and healing properties of the mixture according to the properties of the ingredients[27,28,29,30,31,32,33,34,35,36,37,38,39,40,41,42,43,44] (see Table 4).

**TABLE 4**
**Some Physical and Biological Properties of the Mixture Ingredients**

| Honey | Olive Oil | Beeswax |
|---|---|---|
| Increases nitric oxide | Increases nitric oxide | Decreases prostaglandins |
| Decreases prostaglandins | Decreases prostaglandins | Antioxidant effect |
| Liberates $H_2O_2$ | Antioxidant effect | Anti-inflammatory effect |
| Acidity effect | Anti-inflammatory effect | Healing property |
| Osmolality effect | Healing property | Decreases Leukotriene B4 |
| Antioxidant effect | Decreases Leukotriene B4 | |
| Anti-inflammatory effect | Modulate cytokines production | |
| Healing property | Antimicrobial effect | |
| Antimicrobial effect | | |
| Modulate cytokines production | | |

Honey has been valued highly in the Middle East for centuries. It was mentioned in the Holy Quran 1,400 years ago (And thy LORD taught the bee to build its cells in hills, on tree and in men's habitations, then to eat of all the produce of the earth and find with skill the spacious paths of its LORD, there issues from within their bodies a drink of varying colors, wherein is healing for men, verily in this is a sign for those who give thought). It is also mentioned in the Holy Talmud. Hippocrates and Celsus used honey for wounds and ulcers. Honey has antibacterial, antifungal, antiviral, and antioxidant activities; it reduces prostaglandins concentration and enhances nitric oxide production. Nitric oxide, which has antimicrobial activity, can accelerate wound healing and improves microcirculation of the flap and increases its survival rates[33,45,46,47]. Exogenous nitric oxide has been shown useful in decreasing the internal anal sphincter tone. Nitric oxide donors have been used for treatment of chronic anal fissure and acute strangulation of prolapsed internal hemorrhoids[48,49,50]. Glyceryl trinitrate ointment has been used for the treatment of chronic anal fissure since it caused relaxation and anal dilation[51]. Prostaglandins, a well-known mediator of inflammation, increased markedly in sensitive and inflammatory skin diseases[52]. Prostaglandins caused vasodilatation and pruritis[53]. The effects of the honey mixture might be due to reduction in the prostaglandin synthesis at the site of application, elevation of nitric oxide in the lesions, inhibition of fungal or bacterial growth, and to its antioxidant and anti-inflammatory activities[17,18,22].

The main limitations of this study are the small number of patients recruited, the type of study (which included a pilot clinical trial), and the lack of measurement of anal pressure. However, this study aimed to provide some evidence of efficacy and rationality to proceed further with randomized controlled studies. If this trial is substantiated by controlled studies, it will pave the way for use of this mixture in the management of anal diseases and inflammation, and also during the postoperative period following surgical intervention. Furthermore, such new intervention might be useful in countries where there is limited access to modern medicine.

## BIOSKETCH

**Noori S. Al-Waili**, **MD, DOG, PhD, CHT**, is Director of Clinical Research, Life Support Technologies INC, Chronic Wound Treatment and Hyperbaric Medicine Center, The Mount Vernon Hospital, Mount Vernon, NY. Dr. Al-Waili is a physician and research scientist who has published more than 160 scientific papers in nephrology, urology, alternative medicine, hypertension, diabetes, malignancy, immunity, and biomedical sciences, including a wide range of new therapeutic measures. He has developed many theories and discoveries in modern and alternative medicine that have been published in esteemed international scientific journals and he has published several exiting papers in nephrology/urology including peritoneal macrophages transfusion, enuresis, renal colic, frequency of micturiton, and more than 30 scientific clinical and laboratory publications on honey. Presently his main interest is focused on nephrology/urology, wound care, and hyperbaric medicine, as well as alternative medicine. Dr. Al-Waili serves as Editorial Board Member of several medical journals, a member of evaluator panel, Current Drug, UK, and a reviewer for many medical and biomedical journals. E-mail: noori786@yahoo.com
